# Task representation and individual differences affect strategy selection and problem-solving performance

**DOI:** 10.3389/fpsyg.2025.1445200

**Published:** 2025-03-18

**Authors:** Xinyu Xie, Jarrod Moss

**Affiliations:** Department of Psychology, Mississippi State University, Mississippi State, MS, United States

**Keywords:** strategy selection, problem solving, working memory capacity, attentional control, task representation, individual differences

## Abstract

**Introduction:**

While strategy selection theories generally posit that people will learn to prefer more successful task strategies, they often neglect to account for the impact of task representation on the strategies that are learned. The Represent-Construct-Choose-Learn (RCCL) theory posits a role for how changing task representations influence the generation of new strategies which in turn affects strategy choices. The goal of this study was to directly replicate and extend the results of one experiment that was conducted to assess the predictions of this theory.

**Methods:**

The predictiveness of a feature of the task was manipulated along with the base rates of success of two task strategies in the Building Sticks Task. A sample of 144 participants completed this task and three individual differences tasks.

**Results:**

The results of the study replicated all prior results including: (1) a salient feature of the task influences people’s initial task representation, (2) people prefer strategies with higher base rates of success under a task representation, (3) people tend to drop features from the task representation that are found not to be useful, and (4) there are more representation and strategy changes when success rates are low. In addition to replication of these findings, individual differences in attentional control, working memory capacity, and inductive reasoning ability were measured and found to be related to BST problem-solving performance and strategy use. Critically, the effect of inductive reasoning and attentional control on solution time was found to be mediated by measures that tap into monitoring of problem attempts and more effective problem space exploration by avoiding repeating past attempts.

**Discussion:**

The results support many of the predictions of RCCL, but they also highlight that other theories may better account for some details.

## 1 Introduction

Problem solving is a ubiquitous human activity that requires individuals to identify a goal and determine the steps needed to achieve it. While there are many different strategies that can be used to solve a problem, individuals vary in the strategies they use (Lemaire and Lecacheur, 2010; Siegler and Lemaire, 1997), raising the question of how people develop and choose the strategies they use. Several theories of strategy selection explain how better performing strategies become preferred (Erev and Barron, 2005; Lieder and Griffiths, 2017; Rieskamp and Otto, 2006; Shrager and Siegler, 1998). Most of these theories start with some kind of task representation and a set of available strategies. However, the Represent-Construct-Choose-Learn (RCCL; Lovett and Schunn, 1999) theory posits a role for how changing task representations influence the generation of new strategies which in turn affects strategy choices. Given this theory’s relatively broad focus on the strategy selection problem from representation to strategy selection, the goal of the current study was to replicate and extend the results of one of only two studies that have been conducted to directly assess the predictions of the RCCL theory.

**Figure 1:**
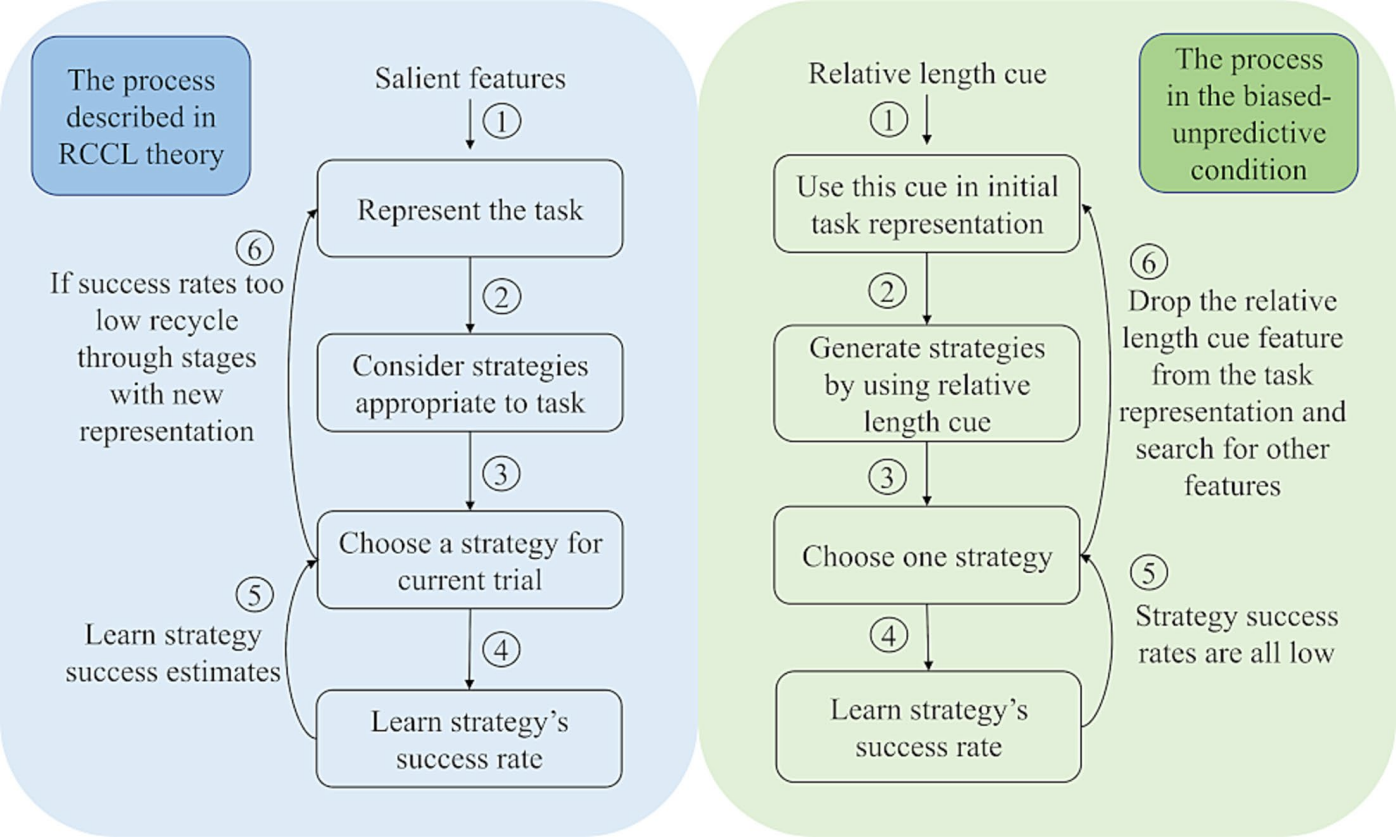
The left side describes the stages of the RCCL theory, and the right side shows how these processes would be instantiated in the biased-unpredictive condition in the current study using the Building Sticks Task, where the relative length cue is not predictive of the correct strategy.

In the RCCL theory, the four main stages are as follows: (1) Represent the task, (2) Construct a set of strategies based on features in the task representation, (3) Choose from among those strategies based on rates of success, and (4) Learn or update success rates based on performance with experience (Lovett and Schunn, 1999). The interaction of the theorized processes is shown in the left half of Figure 1. This theory describes the process of how people use their task representations to learn to make choices and why people might change their task representations over time. A task representation in this theory is defined as a set of features that is used to encode the task environment. The salience of the features in a task impacts the initial task representation by determining which features might be initially selected. Then the selected features of the task representation are used to generate different strategies for use. After constructing a set of strategies, individuals choose a strategy among them based on the strategy’s estimated success rate. The success rate of each strategy is learned from experience in the task. With more experience, people will learn the success rates of all strategies they have used in a task. This learning mechanism leads to gradual changes of the estimated success rate, and these changes in turn lead to strategy selection changes. In particular, the theory says that if all strategies available under the current task representation have low success rates, then individuals seek other features to re-represent the task by adding or removing task features from the representation. New strategies will be generated from this revised representation and their success rates will be learned through experience. Critically, only strategy-specific success rates are learned, and these strategies are based on the features incorporated into the task representation at the time the strategies are created.

One reason why the RCCL theory focuses on the role of task representations in strategy selection is because it was developed, in part, to explain why people exhibit base-rate neglect on some tasks but not others. Lovett and Schunn (1999) argue that the tendency to neglect base-rate information is due to the way people represent these tasks. RCCL explains that strategies are based on the task representation, and people can only learn base rates for strategy success. If the task representation does not include a feature critical for learning base rates in the task, then people will fail to learn these base rates because both the initial and any subsequent strategies are based on the task representation.

No other theory of strategy selection that we are aware of incorporates the role of a changing task representation in creating and selecting strategies. Many other strategy selection theories focus primarily on learning which pre-existing strategy performs better on average (Erev and Barron, 2005; Rieskamp and Otto, 2006). The SCADS theory of strategy selection (Shrager and Siegler, 1998) also incorporates strategy generation mechanisms, but it assumes that the task representation does not change. The Rational Metareasoning (RM) theory (Lieder and Griffiths, 2017) provides an explanation for how people can adjust their strategy selection based on the feature-based representation of individual problems based on success rates and the execution time of strategies on prior problems with similar features. The RCCL theory posits that people will develop an initial task representation from the available task features, generate strategies based on those features, and drop features from the representation that are not useful, and that strategies based on the current task representation are the only ones that will be selected and whose estimates of success will be updated. However, unlike SCADS or RM, RCCL is not a running mathematical or computational model. If the predictions that RCCL makes have empirical support, then it may be worth further developing this theory due to its focus on the importance of task representation changes impacting the generation and availability of strategies that can be selected. The original paper supporting the RCCL theory provided support from two studies (Lovett and Schunn, 1999), but neither study appears to have been replicated since the original publication, nor does it appear that the predictions of this theory have been tested in other studies examining strategy selection.

**Figure 2:**
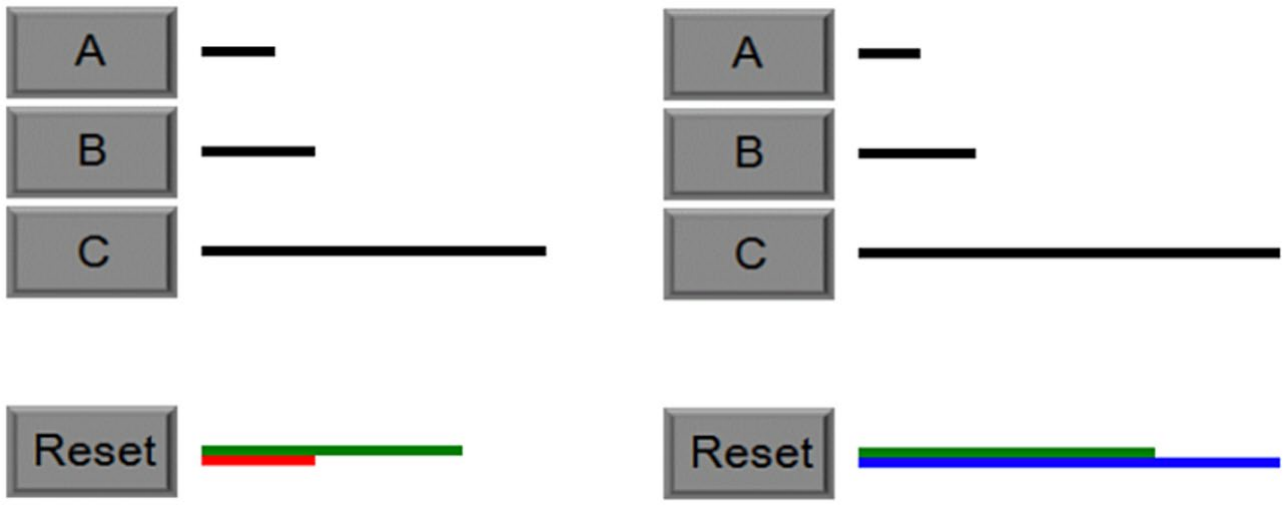
Examples of the undershoot Strategy (left) and the overshoot strategy (right). Next to each building stick is a button (i.e., A, B, C). To add or subtract to the target stick length, participants simply click the button next to the stick. Sticks can be selected multiples times. These two BST problems give an example of building a stick starting with B or C, respectively. If the stick being built is shorter than the target stick, its color will be red (left), and the next selected stick will be added to the stick being built. If the stick being built is longer than the target stick, its color will be blue (right), and the next selected stick will be subtracted from the stick being built. When the stick being built is that same length as the target, the problem is done, and the next problem is presented. While solving a problem, the reset button will erase the stick being built so that a new attempt can be started.

Some of the predictions of the theory can be illustrated using the Building Sticks Task (BST) that was used in the first study of the original paper (Lovett and Schunn, 1999). As shown in Figure 2, in a BST problem there are three building sticks (black) and a target stick (green). The goal of the task is to add and subtract the lengths of the three sticks to match the target length. A participant’s strategy can be categorized as either the undershoot or overshoot strategy based on the first move made on each problem attempt. The undershoot strategy starts with the longest stick that is shorter than the target (i.e., stick B) and then adds additional sticks to reach the target length (as shown on the left half of Figure 2). In contrast, the overshoot strategy starts with the longest stick (i.e., stick C) and then subtracts other sticks to reach the target length (as shown on the left half of Figure 2). In either case, after the first move, additional moves must be made to solve the problem. For the problem shown in Figure 2, the correct strategy is overshoot, and after the first move, subtracting stick A twice from the blue stick will make it match the length of the green target stick.

The original study manipulated the predictiveness of one feature of the task in addition to manipulating the overall base rates of success for the overshoot and undershoot strategies. Specifically, the proportion of problems solved by one strategy was varied to manipulate the base rates of strategy success across conditions to create a biased condition (70% of problems solved by one strategy) or an unbiased condition (50% solved by each strategy). In BST problems, there is usually one stick closest to the target length, which is a salient feature of the task for participants, and they will select this stick in accordance with a hill-climbing heuristic (i.e., choosing moves based on which one decreases the distance to the goal the most; Lovett and Anderson, 1996). For example, in Figure 2, stick C is closer to the target than stick B, so participants using the relative length cue would be more likely to use the overshoot strategy. However, using the relative length cue may or may not be the correct strategy for a given BST problem, and the success rate of the relative length cue was also manipulated as a second factor in the original study to create a predictive condition (80% predictive of a correct strategy) or an unpredictive condition (50% predictive of a correct strategy).

To illustrate how the task representation affects strategy selection, consider a participant in the biased-unpredictive condition that starts off by using the relative length cue as a task feature in generating a strategy. The strategies generated based on this feature would be in the format of if-then production rules according to RCCL. An example of such an overshoot strategy would be if the overshoot stick is much closer to the goal than the undershoot stick, then select the overshoot stick and try to reduce its length to match the target. An equivalent strategy could be constructed for undershoot as well from this feature. In general, these strategies will turn out to be unsuccessful (50% success rate), and therefore the participant drops the relative length cue feature from the task representation according to the RCCL theory. The participant may identify other features of the task to help select between overshoot and undershoot, but eventually all unsuccessful features would be dropped. In this case, the only remaining strategies are to choose the overshoot or undershoot strategy without using any problem-specific features. At this point, the participant would learn the overall base rates of success for these two strategies resulting in choosing the strategy with the highest base rate most often. This process is illustrated in the context of the RCCL theory on the right side of Figure 1.

As an alternative to a discrete representation change like that posited by RCCL, the RM framework would suggest that the relative length cue could become less important to selection of existing strategies over time as learning occurs. The data and figures reported from the original study (Lovett and Schunn, 1999) make it difficult to assess whether a gradual shift has occurred or a discrete change has occurred when the relative length cue is unpredictive. An additional goal of the current study was to examine the data in more detail to see if evidence of a discrete shift was present.

With the manipulations of base rates and cue predictiveness, RCCL’s predictions are as follows: (1) the relative length cue will influence participants’ initial strategy selection because salient cues are more likely to be a part of the initial problem representation; (2) participants will eventually learn the base rates of success of each strategy in the biased conditions; (3) participants will stop using the relative length cue feature if it is not predictive of success; (4) more task representation and strategy changes will occur when the success rates of strategies are low. The relationship between strategy selection and problem representation in the RCCL theory also suggests possible links between individual differences that affect how people construct problem representations and select strategies.

## 2 Individual differences related to problem solving and strategy selection

Individual differences in working memory capacity as well as attentional control processes are likely to affect strategy selection and problem-solving success. Working memory capacity has previously been linked to success in problem solving (Ash and Wiley, 2006; Wiley and Jarosz, 2012) at least partially because of its association with attentional control ability (Ash and Wiley, 2006; Draheim et al., 2021; Engle and Kane, 2004; Kane et al., 2004). Attentional control is thought to be involved in selecting which representations to maintain in working memory (Unsworth, 2016; Unsworth et al., 2021). In addition, considering that generating the initial task representation requires attention to feature saliency, strategy selection might be correlated with individual differences in the ability to control attention. Attentional control may be an important aspect of both problem-solving and strategy selection processes because it allows people to focus on relevant information and filter out distracting information.

Previous research examining strategy selection and individual differences has identified both working memory capacity and inductive reasoning as individual differences that are associated with strategy selection (Schunn et al., 2001; Schunn and Reder, 2001). In a task, inductive reasoning can play a role in finding an underlying pattern, which may drive both representation change and strategy selection to take advantage of this pattern. People with higher working memory capacity are able to hold more relevant information in working memory, which in turn can help them to choose correct strategies (Schunn and Reder, 2001). However, other work found there was not a relationship between working memory capacity and strategy selection, although both working memory capacity and inductive reasoning were related to the awareness of base rate changes in the task (Schunn et al., 2001). The inconsistent findings on whether working memory capacity is related to strategy selection may be due to several factors, including differences in the tasks used to measure working memory capacity and the complexity of the problems being solved. One question is whether updating strategy success estimates is implicit or explicit. If the learning is implicit, it may not be affected by working memory capacity and inductive reasoning because implicit learning processes such as reinforcement learning may be governed by different neural systems than those associated with working memory (Collins and Frank, 2012). However, if learning strategy success rates is explicit, it is more likely to be associated with working memory capacity and inductive reasoning ability. Exploring these relationships can help clarify the underlying mechanisms involved in strategy selection and problem-solving.

Although individual differences in working memory capacity, attentional control, and inductive reasoning ability could be related to strategy selection processes, it is also important to understand the relationship of these individual differences to the problem-solving task itself. Doing so means that individual differences associated with task performance can be considered when examining the relationship of these individual differences with strategy selection in the task. For example, if working memory capacity is related to performing one of the strategies because it is more demanding on working memory resources, then individuals with lower working memory capacity may select other strategies that have lower success rates but that demand less of working memory (Beilock and DeCaro, 2007). Given the larger sample size required for examining individual differences and the four between-participant conditions in the original study, in the current study we chose to focus on primarily examining individual differences related to BST problem solving. The BST is a problem-solving task that allows individuals to experience multiple attempts on a single problem as they search for the correct solution. In addition, since most problem-solving actions are captured via the computer interface to the BST, it is possible to examine exploration of the problem space and its association with the overall outcome (i.e., time to solve) with respect to individual differences. Prior work has shown relationships between individual differences, such as working memory capacity, and time to solve a problem (Ash and Wiley, 2006), but in the current study we examine more fine-grained measures of problem space exploration to examine whether these measures mediate the relationship between individual differences and solution time.

After selection of the initial move on a BST problem, the problem solver is either trying to add additional sticks to the first stick or to subtract sticks from the first stick. Each move involves a comparison of stick lengths to the difference between the target and current stick length. Either this attempt will succeed, the problem solver will eventually decide to reset the problem and start again, or a move limit is reached (set to six moves in the current study) and the task resets itself. Strategy selection will occur again followed by a similar set of moves and further problem attempts until the problem is solved. The current study explores whether measures of attentional control, inductive reasoning ability, and working memory capacity are related to this problem-solving process.

While these analyses were exploratory in nature, these measures were selected based on their prior use in problem-solving and strategy research and because of their plausible role in supporting the BST problem-solving process. For example, inductive reasoning and working memory capacity may affect an individual’s ability to reason about multiple moves in a BST problem before selecting a move. Reasoning ability and working memory capacity may also play a role in reasoning about the way in which prior attempts have failed when selecting moves on a subsequent problem attempt with working memory playing a role in how well people can maintain and access traces of prior attempts. Attentional control and working memory capacity could also play a role in maintaining a move count to assess progress on the problem and how close one is to the move limit.

In this study, we aim to replicate one of the studies that is the primary support for the RCCL theory and examine its predictions about how features in the problem environment and base-rate learning influence strategy selection. In addition to replicating the original analyses, the data can be used to examine whether there is evidence of a discrete representation change. We also extend this previous work to investigate whether individual differences in attentional control, inductive reasoning ability, and working memory capacity are related to BST problem solving and the use of the two primary strategies.

## 3 Materials and methods

### 3.1 Design

A 2 (relative length cue predictiveness: predictive – 80%/20%; unpredictive – 50%/50%) by 2 (base rate bias: biased – 70%/30%; unbiased – 50%/50%) design was used in this experiment. The study consisted of a pretest, training, and posttest phase. The pretest and posttest phases are designed to probe strategy preferences and are identical across all conditions. The four experimental conditions only differed in the set of training BST problems that participants solved in the training phase. Table 1 shows the different proportion of problem types in each condition.

**Table 1:** The proportion of problem types in each of the four conditions.

Condition	UC/US	UC/OS	OC/OS	OC/US
Unbiased-unpredictive	0.25	0.25	0.25	0.25
Unbiased-predictive	0.40	0.10	0.40	0.10
Biased-unpredictive	0.35	0.15	0.15	0.35
Biased-predictive	0.48	0.02	0.30	0.20

UC (undershoot closer) means that the undershoot stick is closest to the target. OC (overshoot closer) means that the overshoot stick is closest to the target. US (undershoot solved) means that the undershoot is the correct strategy. OS (overshoot solved) means that the overshoot is the correct strategy. This table shows the design for the case when the undershoot strategy was designated as the biased strategy, but which of the two strategies was selected as the biased strategy was counterbalanced across participants. A slight difference from the specified proportion of predictiveness and base rates in the biased-predictive condition was used so that each condition had at least one problem for each problem type.

In the biased base rate conditions shown in Table 1, undershoot is the more successful strategy with 70% of the training problems being solved by undershoot. In the experiment, which of the two strategies had the higher base rate of success was counterbalanced across participants. So there actually were 8 experimental conditions when including this counterbalancing. For the unbiased conditions, which strategy was designated as the most successful strategy was also counterbalanced although this did not alter the composition of the training problems because the bast rates for each strategy were 50%. However, the designation of which strategy was most successful was used during analysis of the data where the primary measure was how often participants selected the most successful strategy based on the condition they were assigned to. Randomly assigning one of the two strategies as the most successful strategy in the unbiased conditions made it possible to collapse across the counterbalancing of overshoot and undershoot to analyze the differences in how often participants selected the most successful strategy for their condition for all four conditions.

A large pool of BST problems consisting of the four problem types shown in the columns of Table 1 was used to select the BST problems that each participant solved during the training phase. When a participant was randomly assigned to one condition, 80 BST problems were selected from the problem pool according to the number of different problem types for that condition as shown in Table 1. For example, if the participant was in the unbiased-predictive condition, 32 UC/US problems, 8 UC/OS problems, 32 OC/OS problems, and 8 OC/US problems were selected from the problem pool. Thus, the BST problem set for each participant is different but has the distribution of problem types specified by the condition. This problem selection process was used to sample relevant BST problems throughout the space of possible BST problems to avoid item-based effects in the data that might be caused by a particular “weird” problem that affects that condition’s mean.

### 3.2 Participants

A sample of 144 undergraduate students from Mississippi State University participated for course credit, and forty-four participants’ data was excluded from analyses because they did not meet the criterion of 80% accuracy on parity judgment in the complex span task (*N* = 38) or never responded correctly in complex span task (*N* = 4) or had more than five-time delays in the letter series task (*N* = 4). These exclusion criteria were established prior to data collection.

A power analysis was conducted based on the results reported by Lovett and Schunn (1999), with the partial eta square being 0.35 (from Experiment 1, ANOVA interaction effect reported on p. 116). This effect was selected because it was one of the smallest effect sizes reported in the paper. With a significance criterion of *α* = 0.05 and power = 0.90, the minimum sample size needed with this effect size is *N* = 98 for detecting this interaction. Thus, the target sample size was set to 100.

The study was reviewed and determined to be exempt by the Mississippi State University Institutional Review Board (protocol number 19–349). Participants provided informed consent via an electronic consent form presented before the study began.

### 3.3 Procedure

In addition to the BST, participants were given three tasks designed to measure individual differences, including the antisaccade task (Kane et al., 2001), a complex span task (Barrouillet et al., 2007), and a letter series completion task (Simon and Kotovsky, 1963). The antisaccade task is designed to measure the ability to control attention, the complex span task was used to measure working memory capacity, and the letter series task measures the ability to acquire concepts by induction from examples, that is inductive reasoning ability. The entire session including all tasks took 70–80 min to complete with up to 6 participants at a time completing the task during a single session. The order of the tasks was the antisaccade task, the BST, the complex span task, and the letter series task. The antisaccade task first because it required monitoring participants to ensure that they were seated at the correct distance from the screen for the visual angle requirements described below for that task. It was easier for the experimenter to monitor this requirement if all participants were completing the antisaccade at the same time. The remainder of the tasks were presented in the same order for each participant so that fatigue effects would be similar across participants. At the beginning of the experiment, participants read the consent form on the computer and then moved to the antisaccade task if they consented.

#### 3.3.1 Anti-saccade

This task assesses the ability of participants to control their attention and not look at a pro-saccade stimulus in order to identify a target stimulus presented on the opposite side of the screen with the task being adapted from Kane et al. (2001). Initially, the participants practiced mapping responses to the keys on the number pad (1, 2, and 3) for the letters B, P, and R, respectively. They had to place their left hand on the spacebar and their right hand on the number pad. A “Ready?” prompt appeared on the screen, and participants started each trial by pressing the spacebar. Then after 400 ms a fixation cross appeared in the center of the screen. To avoid cue onset familiarity, fixation times were randomly selected from 200 ms, 600 ms, 1,000 ms, 1,400 ms, 1800 ms, or 2,200 ms durations. Then the target letter was displayed for 100 ms and then replaced by a mask letter ‘H’ for 50 ms, followed by the number ‘8’ as a second mask that would remain on the screen until participants made a response. Participants would hear an audio tone for incorrect responses. There were 18 trials in the practice block. Participants had to reach 80% correct or higher during the practice block in order to proceed, otherwise, the practice block would continue in a loop until the criterion was met or participants completed 10 times. No participant failed to meet this criterion.

**Figure 3:**
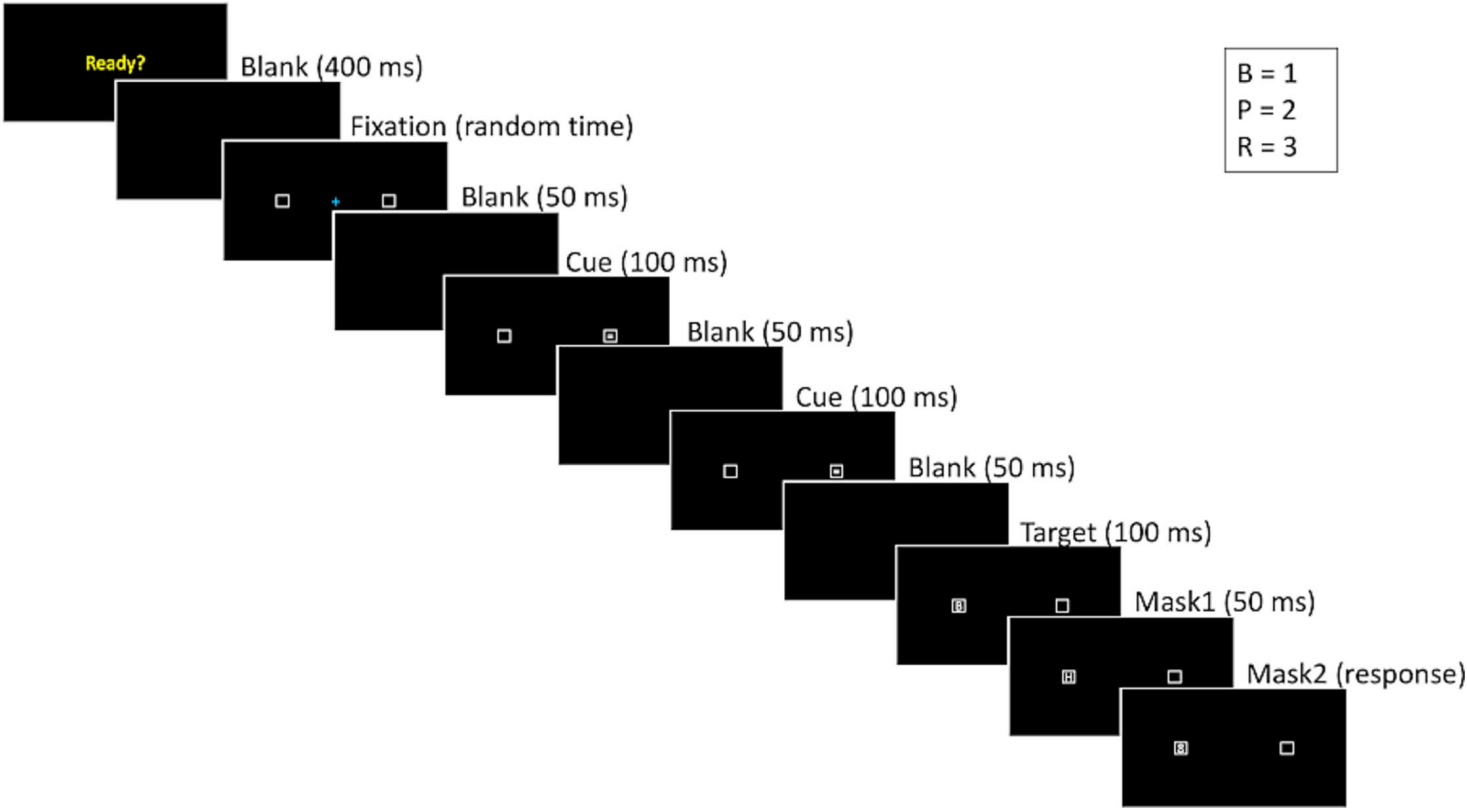
The structure and timing of an antisaccade trial.

After practicing the response mappings, participants received instructions on the antisaccade trials and completed 18 warmup trials and 36 actual antisaccade trials. These trials were presented as a single block of 54 trials. The antisaccade trials followed a similar procedure as the response mapping trials that is shown in Figure 3, with a “Ready?” prompt, a fixation cross, and presentations of the pro-saccade cue and anti-saccade target. The pro-saccade cue and anti-saccade target appeared in opposite flanking positions, and the target was replaced by two masks (letter ‘H’ followed by number ‘8’) that remained on the screen until the participant made a response. The target appeared on each half of the screen an equal number of times. The distance between the fixation and the closest edge of each square where the target and cue appeared spanned 11.5 degrees of visual angle. An audio tone was played for incorrect responses. The dependent measure was the proportion of trials in which the participant correctly identified the target letter.

#### 3.3.2 Building sticks task

Instructions for how to solve the problems by overshoot and undershoot strategy were shown on the computer, so that participants were aware of the two basic approaches to solving BST problems. The strategies were not given explicit names (e.g., undershoot) in the instructions. Participants completed a set of three practice problems. For the first practice problem, they were told to use start with the stick that was longer than the target (overshoot). For the second practice problem, they were told to use start with a stick that was shorter than the target (undershoot). For the third problem, they were not told to use a specific approach so that they had to figure out for themselves which of the strategies solved the problem (the problem was solved by undershoot).

The task has a pretest, training, and posttest phase. The pre-and posttest phases are a set of 10 test problems before and after the training phase in which participants only select the first move that they would make in attempting to solve it. The purpose of this format is to assess strategy performance without providing feedback about the success of the strategy. For these test problems, there were five levels of the relative length cue that were each sampled twice. There were two problems with a strong cue toward the undershoot strategy, two problems with a weak cue toward the undershoot strategy, two neutral problems (two sticks were equally close to the target length), two problems with a weak cue toward the overshoot strategy, and two problems with a strong cue toward overshoot the strategy. The distinction between a strong cue and a weak cue was based on the ratio of difference between stick B and the target stick to difference between stick C and the target stick or reversely. Specifically, if the ratio was greater than 3.5, then the cue was considered strong, while if the ratio was around 2, then the cue was considered weak. During the training phase, participants were given 80 training problems based on the condition they were assigned to as shown in Table 1. Participants were required to work on each of these problems until the length of the stick they were building matched the length of the desired stick or they used a “reset” button to start a new solution attempt. When a problem was presented, participants can click on each of the three available sticks to add it to the current attempt. Each stick can be selected as many times as needed. If the current attempt is longer than the target, then clicking on a building stick will subtract that stick from the current attempt. If the current attempt is shorter than a target, then the clicked stick is added to the current attempt. After each addition or subtraction, the current attempt stick is updated on that task interface.

Participants were told that all problems can be solved in 6 moves or less. The experiment automatically reset the problem after 6 moves (on their seventh move). At the end of the task, participants were asked to answer three questions: “How did you decide which stick to use on your first attempt at the problem?,” “Did you use any particular strategies to solve these problems?,” “Were there any strategies you tried that did not seem to work? If so, please describe what you tried.” Participants were not provided any instructions about proceeding as quickly as possible or to try to solve the problem in as few attempts as possible.

#### 3.3.3 Complex span task

Next, participants completed a modified version of the complex span task used by Barrouillet et al. (2007). First, participants were asked to complete a training phase in which they would make judgments on the parity of the numbers 1–10. They had to achieve 80% accuracy to continue, otherwise, they would repeat the training. In the main task, participants were presented with a series of letters ranging in length from one to seven, with three series of each length. The series were presented in ascending order with all the one-length series presented before moving on to the two-length series and so on. The 21 series contained a total of 84 letters, each followed by a series of 4, 6, or 8 integers from 1 to 10. The numbers appeared in a fixed random order, with as many even as odd numbers. Participants were required to remember the letters in order and judge the parity for digits. After “Recall” appeared on the screen, participants would press the keys corresponding to the letters they were to remember. Before the main task began, one example of how to respond to digits and recall a letter was shown to participants. Participants were instructed and prompted to keep parity judgment at or above 80% accuracy in the main trials (which was established as a data exclusion criterion prior to the study). Feedback on accuracy was presented after recall for each series of letters.

#### 3.3.4 Letter series task

Finally, participants completed the letter series task (Simon and Kotovsky, 1963). Two examples of letter series with one rule and two rules were presented, and participants were presented with the letter series, rule(s), and answer for these examples. Then a practice trial was provided (e.g., “B, V, D, X, F, Z, H, B? “), and participants were guided to find the pattern within the letters. They were given feedback and taken back re-read the instructions if they did not respond correctly to this practice trial. There were 15 trials. The letters in each trial change according to a fixed pattern which could include more than one rule (e.g., “V, V, F, X, X, H, Z, Z, J, B, B, L, D, D?”). Participants were required to complete each trial in 1 min or less. Participants were not able to go back once they submitted an answer. If they responded too quickly (less than 10 s) and gave a wrong answer, a compulsory delay of 15 s would be triggered. Once participants triggered the delay more than 5 times, their data were excluded from data analyses. The delay was included to discourage answering quickly without attempting to figure out the answer.

## 4 Results

### 4.1 Data analysis

For the pre- and posttest data, participants only selected the first move. Analyses of these data focused on whether participants selected the move consistent with the overshoot or undershoot strategy. Problems in which participants selected stick A were not included in the analyses (2.8% of the data) because stick A was not considered to be using the undershoot strategy as described in the task instructions. Based on the base-rate bias condition to which participants were assigned, one of the two strategies was designated as the successful strategy. In the biased conditions, half of the participants were randomly assigned to experience more problems solved by undershoot, and the other half experienced more problems solved by overshoot. Therefore, the successful strategy for these two groups of participants was undershoot and overshoot, respectively. In the unbiased conditions, participants experienced half of the problems solved by each strategy. In this case, half of the participants were randomly assigned to have the undershoot strategy designated as the successful strategy, and the other half were assigned the overshoot strategy as the successful strategy. Coding the data in this manner made it possible to analyze the data across counterbalance conditions and is identical to how the data were analyzed in the original study (Lovett and Schunn, 1999). For example, it is possible to assess whether participants selected the strategy for which the base rate was higher in the biased condition without regard to whether the strategy with the higher base rate was undershoot or overshoot.

A separate set of analyses used the training phase data, during which participants had to solve each problem to move on to the next problem. Because it may take multiple attempts to solve a problem, it is possible to examine how many of the attempts that participants made were unique (as opposed to repeating a series of moves that was tried previously on that problem). In addition, unsuccessful attempts can be terminated by the participant clicking the reset button (i.e., manual reset) or by making more than 6 moves resulting in the system forcing the problem to be reset (i.e., forced reset). For the training phase data, dependent measures include the strategy that was first selected, solution time, proportion of the attempts that were unique, and the number of forced and manual resets.

The data were analyzed using generalized linear mixed effects models including participant and item random effects unless otherwise noted. Analyses were performed in R using the lme4 and lmerTest packages (Bates et al., 2015; Kuznetsova et al., 2017). The lmerTest package provides *p*-values for fixed effects based on the Satterthwaite approximation. Factor variables were coded using deviation contrast coding (e.g., −0.5/0.5). For all models, a maximal random effects model was initially used (Barr et al., 2013), with participant random intercepts and any within-participant conditions modeled as random slopes for participant. If the model did not converge, then the random effects structure was reduced. While the power analysis determining sample size was based on an ANOVA as was used in the original paper, generalized linear mixed effects models were used because it is possible to formulate models that generally test the same effects as the original ANOVAs while also better representing that in many cases, the dependent measure is a binary outcome (e.g., was a certain strategy selected). In many cases in the original paper, a proportion measure was used in an ANOVA as if it were a continuous response.

The high rate of exclusion for the complex span task (*N* = 38) is discussed in the supplementary analyses available at https://osf.io/8w4kd. Analyses with these participants included are presented there, and in all cases, there is no difference in the statistical significance of the results reported here with these participants excluded from analyses.

### 4.2 Replication of original findings

#### 4.2.1 Sensitivity to cue predictiveness

**Figure 4:**
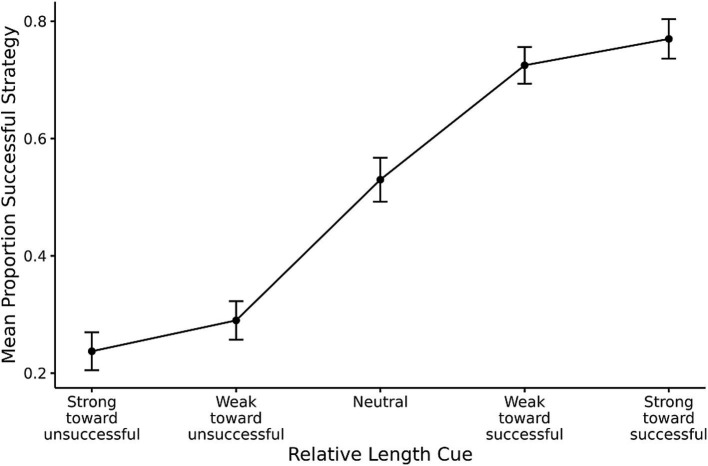
Mean proportion of successful strategy selection based on the relative length cue during pretest. Error bars show one standard error of the mean.

The first prediction from RCCL was that the relative length cue would influence participants’ initial strategy selection. In the BST, the stick that is closest to the target is a salient cue associated with strategy selection. As described, there were five types of problems on the pre- and posttest: strong cue toward overshoot, weak cue toward overshoot, neutral, weak cue toward undershoot, and strong cue toward overshoot. These problem types were recoded based on the most successful strategy as designated by the condition the participant was assigned to (e.g., strong cue toward the most successful strategy). For the pretest, if participants were sensitive to the length cue, there would be a higher proportion of successful strategy selection for those problems in which there is a more salient relative length cue predictive of a successful strategy. If not, the proportion of successful strategy selection would be a flat line around 50%. The mean proportion of the time that the successful strategy was selected is shown in Figure 4 where it can be seen that participants were sensitive to the length cue in the predicted manner.

The trial-level data were analyzed in a logistic generalized linear mixed effects model, with the dependent measure being whether the successful strategy was selected for each problem. The relative length cue was coded from-2 = strong cue toward unsuccessful strategy to 2 = strong cue toward successful strategy. The generalized linear mixed model showed that the relative length cue had a positive effect on the proportion of successful strategy [*b* = 1.03, *SE* = 0.29, 95% CI (0.46, 1.61), *z* = 3.52, *p* < 0.001], which is consistent with the original finding. Increasing one unit on the relative length cue scale therefore results in a 2.80 increase in the odds of selecting the successful strategy.

#### 4.2.2 Sensitivity to base rates

**Figure 5:**
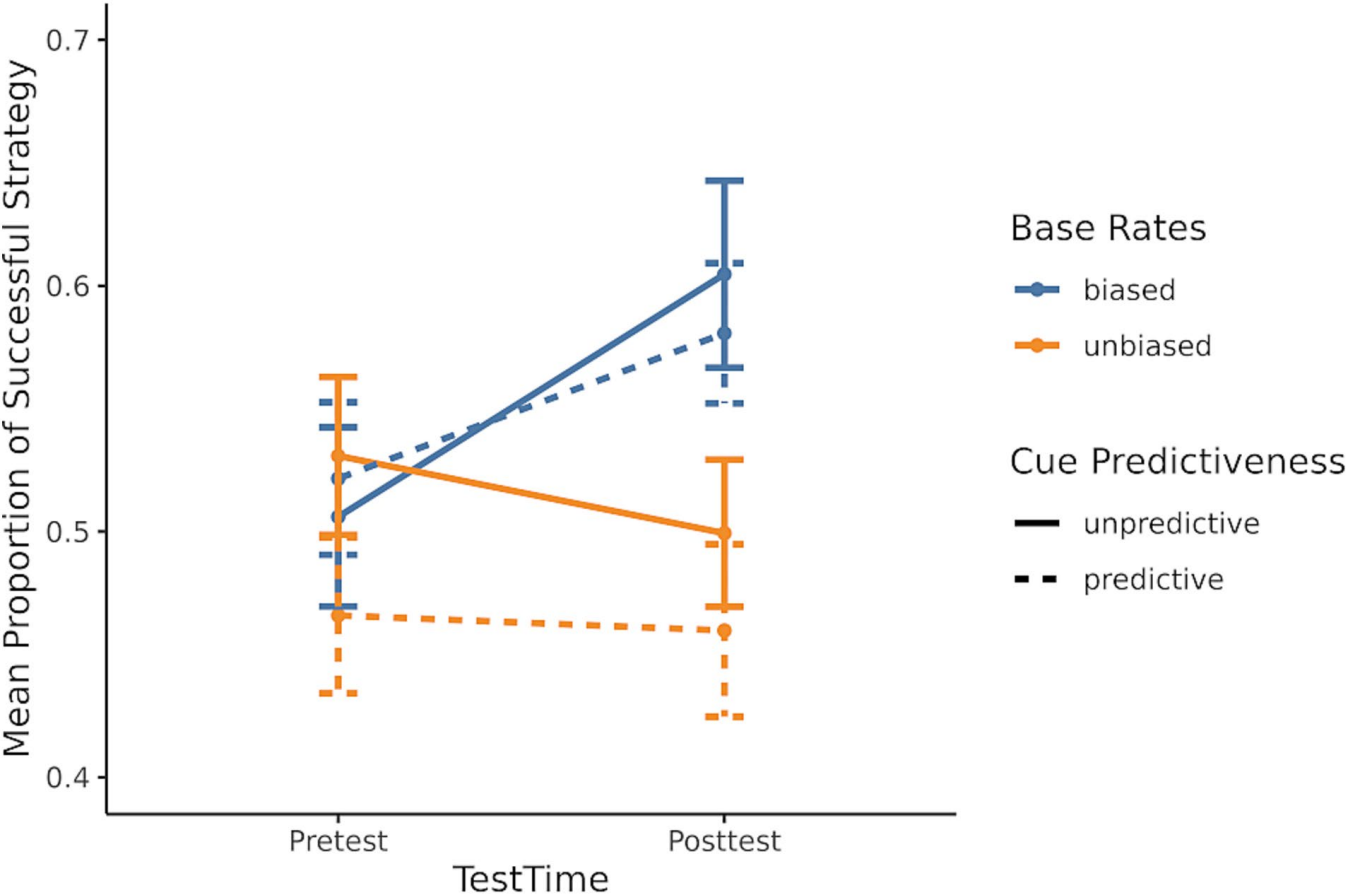
Mean proportion of successful strategy selection for each of the four conditions.

The second prediction was that participants would learn the base rates of success of each strategy. If participants learned the biased base rate during training, they would show sensitivity to the base rates in their strategy selection on the posttest. The biased base rate conditions were compared to the unbiased base rate conditions to test if biased base rates influenced successful strategy selection. To examine this prediction, a generalized linear mixed model was used with the dependent measure being whether the most successful strategy was selected, and the predictors included the base rate bias condition (unbiased/biased), the cue predictiveness condition (unpredictive/predictive), and test time (pre/posttest). The model fit is shown in Table 2, and the results demonstrated that participants in the biased base rates conditions chose the biased strategy more often than those in the unbiased conditions. This result is also consistent with the original findings. Critically, the differences between the base rate bias conditions (unbiased/biased) in the posttest were not caused by differences in pretest strategy selection because there is an interaction between the bias condition and test time. This interaction is driven by there being no difference in successful strategy selection at pretest between the biased and unbiased conditions (*b* = 0.07, *SE* = 0.15, *z* = 0.45, *p* = 0.97), but at posttest, the biased condition had a higher rate of successful strategy selection than the unbiased condition (*b* = 0.46, *SE* = 0.15, *z* = 3.16, *p* = 0.009). This interaction is shown in Figure 5, indicating that participants learned the base rates during training.

**Table 2:** Generalized linear mixed effects model fixed effects for the proportion of successful strategy selection.

Predictors	*b*	*SE*	*z*	*p*
(Intercept)	0.09	0.06	1.50	0.13
Base rate bias	0.26	0.11	2.33	**0.02** ^*^
Cue predictiveness	−0.13	0.11	−1.11	0.27
Test time	0.13	0.10	1.25	0.21
Base rate bias: cue predictiveness	0.19	0.23	0.84	0.40
Base rate bias: test time	0.39	0.19	2.13	**0.03** ^*^
Cue predictiveness: test time	−0.02	0.19	−0.13	0.90
Base rate bias: cue predictiveness: test time	−0.27	0.37	−0.72	0.47

For the model, base rate bias includes the biased base rates condition (coded as 0.5) and the unbiased base rates condition (coded as-0.5), cue predictiveness includes the predictive (coded as 0.5) and unpredictive cue conditions (coded as-0.5), and test time includes the pretest (coded as-0.5) and posttest (coded as 0.5). Bolded entries with * denote values significant at the *p* < 0.05 level.

#### 4.2.3 Irrelevant cues are dropped

**Figure 6:**
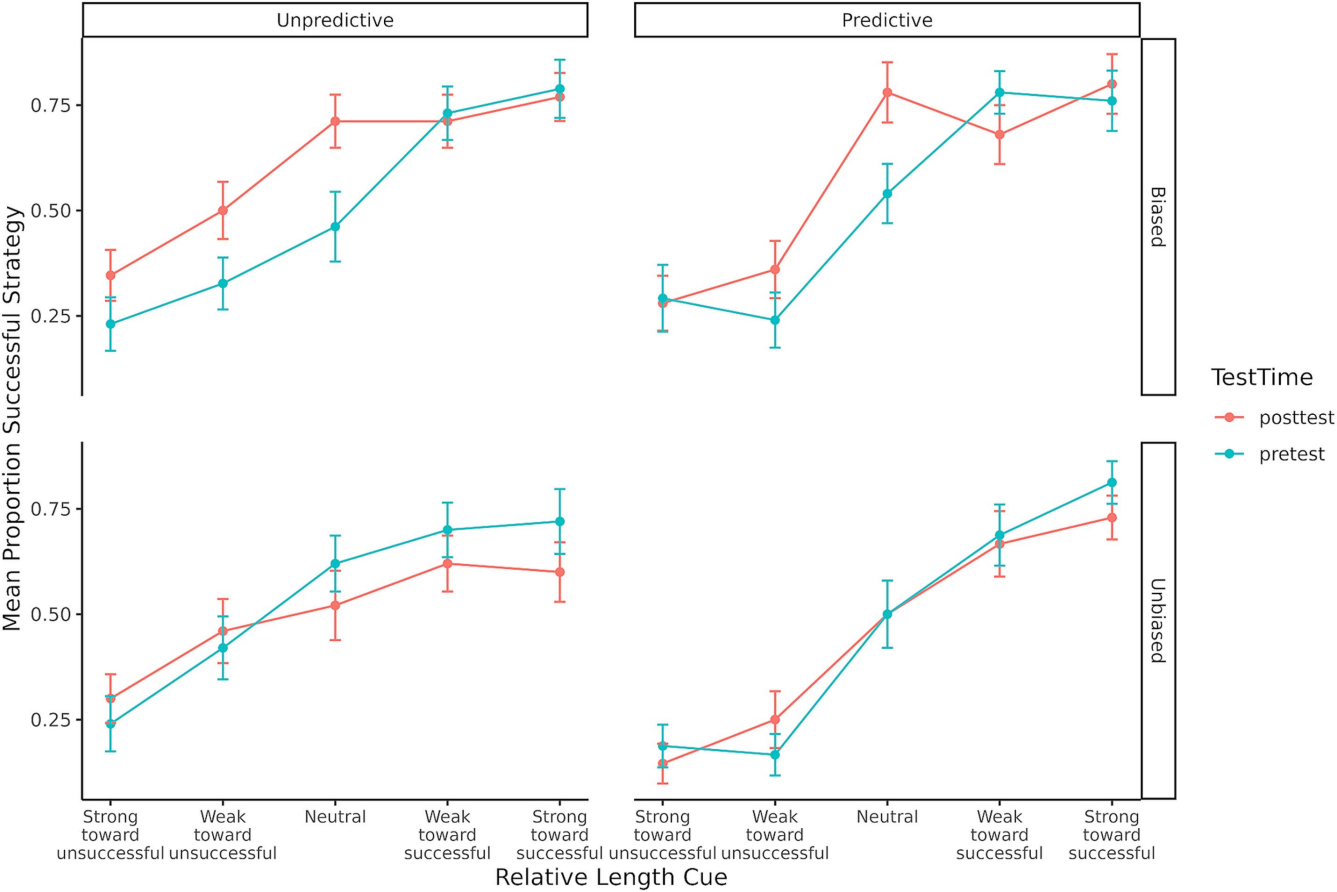
Mean proportion of successful strategy selection for each condition showing the change from pretest to posttest on the slope of effect of the relative length cue.

The third prediction was that people would stop using task features that do not lead to successful strategies. According to the RCCL theory, if one feature was not useful, participants should drop it from their task representation. Consistent with this idea, participants were initially sensitive to the relative length cue during the pretest (see Figure 4). However, those in the unpredictive conditions—where the length cue did not reliably predict success—would become less sensitive to that cue by the posttest. To test this, we fit a generalized linear mixed model of whether the successful strategy was selected during the posttest with predictors of cue predictiveness, base rate bias, and the relative length cue (mean-centered). The non-significant three-way interaction was dropped to aid in interpretation of lower-order effects and the final model is shown in Table 3. Specifically, the interaction between the relative length cue and whether that cue was predictive shows that the slope was greater in the predictive conditions than in the unpredictive conditions. As illustrated in Figure 6, these results support the idea that participants selectively drop unhelpful cues over time.

**Table 3:** Generalized linear mixed effects model fixed effects for the proportion of successful strategy selection at post-test.

Predictors	*b*	*SE*	*z*	*p*
(Intercept)	0.23	0.14	1.62	0.11
Base rate bias	0.67	0.21	3.26	**0.001** ^*^
Cue predictiveness	−0.24	0.16	−1.52	0.13
Relative length cue	0.81	0.33	2.43	0.02
Base rate bias: cue predictiveness	0.16	0.32	0.51	0.61
Base rate bias: relative length cue	0.01	0.16	0.04	0.97
Cue predictiveness: relative length cue	0.47	0.16	2.92	**0.003** ^*^

For the model, base rate bias includes the biased base rates condition (coded as 0.5) and the unbiased base rates condition (coded as-0.5), cue predictiveness includes the predictive (coded as 0.5) and unpredictive cue conditions (coded as-0.5), and the relative length cue is coded as (−2, −1, 0, 1, 2) for strong toward the unsuccessful strategy to strong toward the successful strategy. Bolded entries with * denote values significant at the *p* < 0.05 level.

**Figure 7:**
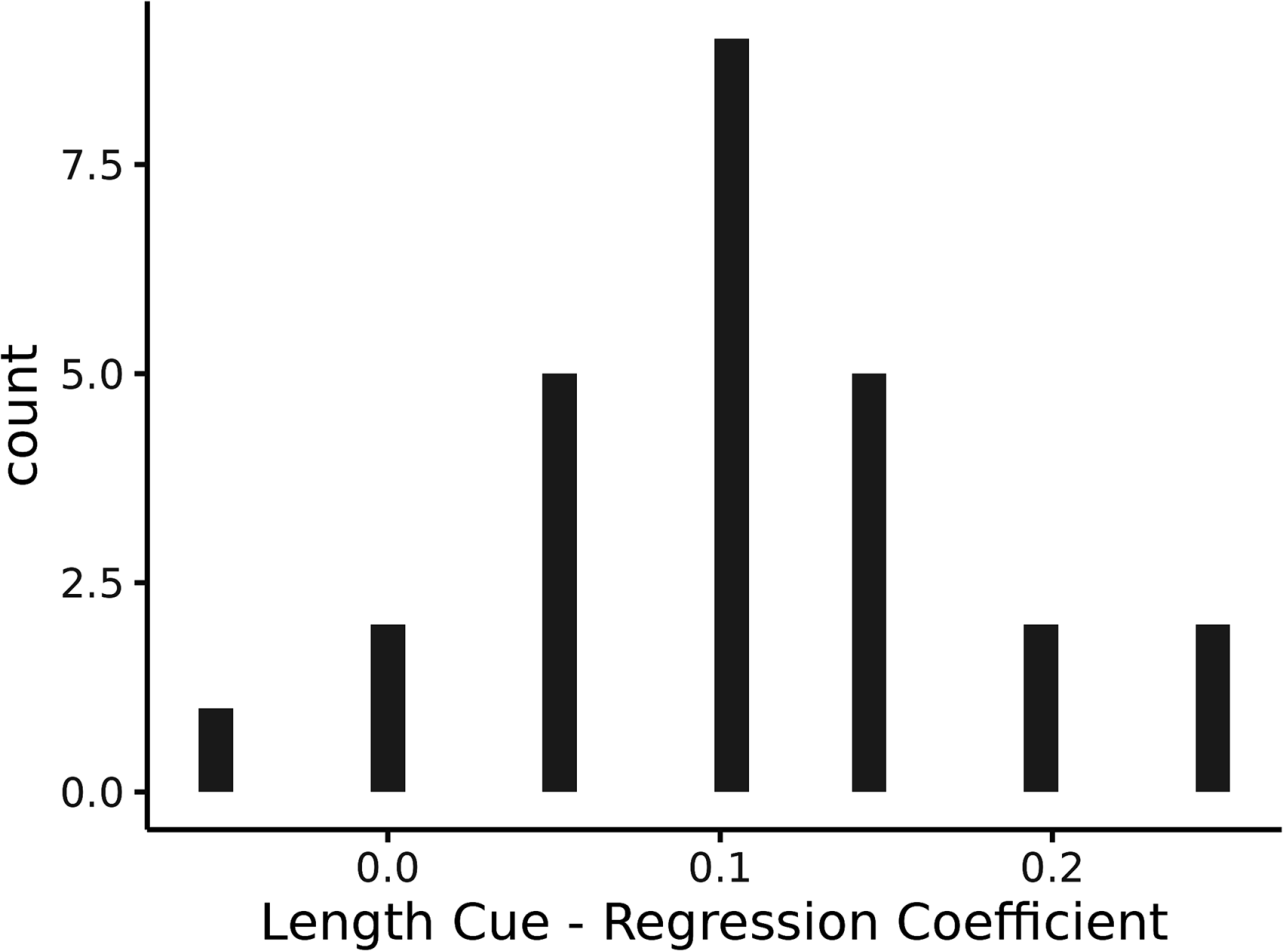
A histogram of the regression coefficients for the cue predictiveness predictor for each participant.

However, if participants had dropped the relative length cue from their representation, then the slope of the posttest line for the unpredictive-biased condition in Figure 6 should have been close to zero. The slope was significantly different from zero (*b* = 0.49, *SE* = 0.10, *z* = 4.86, *p* < 0.001), indicating that on average participants were still sensitive to the relative length cue. In order to examine whether this mean performance was a combination of some participants showing no sensitivity to the relative length cue and others still using the cue, a regression for each participant was performed using the relative length cue to predict the proportion of selecting the successful strategy. A histogram of the regression coefficients for the relative length cue predictor (i.e., slope of the line) for each participant is shown in Figure 7. A bimodal distribution with some participants showing a slope centered around 0 and others showing a positive slope may indicate a mixture of participants who had dropped the cue and others who had not, but the distribution appears to be approximately a unimodal gaussian and centered on a positive slope around 0.1. The mean performance shown in Figure 6 for this unpredictive-biased condition therefore seems to be representative of participants’ strategy selection.

In addition to the posttest data, the responses to the questions about strategies asked at the end of the BST were coded according to the same coding scheme as in the original paper (Lovett and Schunn, 1999). Based on the keywords in the responses, the responses were categorized into three major groups: Length, Exclusive, and Other. Participants who made comparisons between Stick B or C and the desired stick were classified as using a length-sensitive procedure (Length). Participants were categorized as using exclusive strategy if a participant said they “always” or “usually” used one single strategy (Exclusive). The remaining responses were categorized as miscellaneous (Other). The inter-rater reliability between two judges was acceptable with a Cohen’s Kappa value of 0.92.

The proportion of responses falling into each of these three categories for each condition is shown in Table 4. As some evidence that participants’ reported strategies corresponded to behavior, participants reporting using the length cue were more likely to select the strategy corresponding to the relative length cue in the training problems relative to the other two categories combined, *F* (1, 98) = 5.45, *p* = 0.02. Participants in the unpredictive cue conditions had significantly fewer responses related to the length cue (45%, averaged over the levels of the base rate bias conditions) than participants in the predictive conditions (69%, averaged over the levels of the base rate bias conditions), *χ*^2^ = 10.79, *p* = 0.001. This result supports the prediction that irrelevant features will be dropped when they are not predictive of success.

**Table 4:** Proportions of report categories in each condition.

Condition	Report category		
	Length	Exclusive	Other
Unbiased-unpredictive	0.48	0.20	0.32
Unbiased-predictive	0.58	0.21	0.21
Biased-unpredictive	0.42	0.16	0.42
Biased-predictive	0.80	0.08	0.12

#### 4.2.4 More representation change

**Figure 8:**
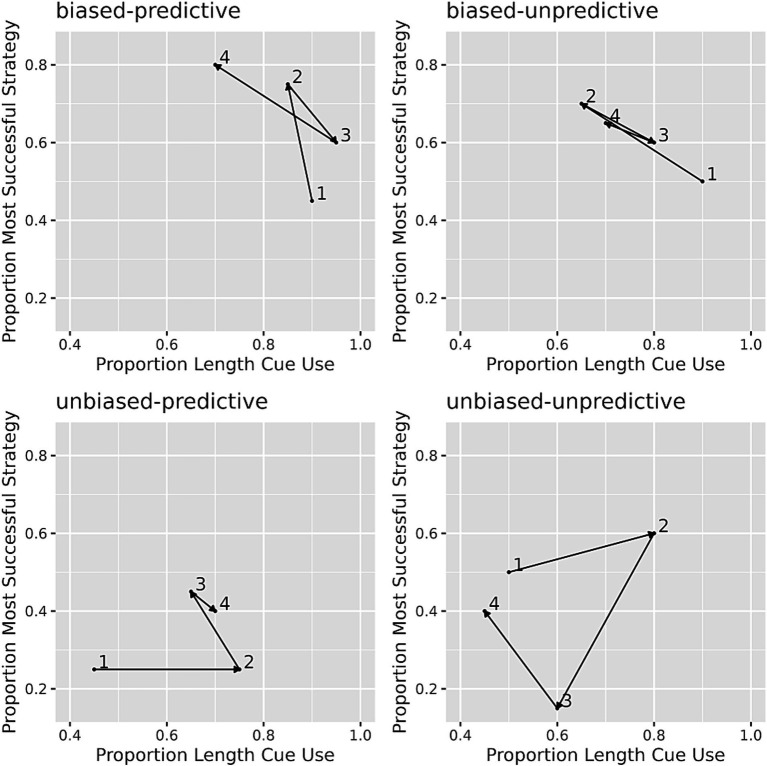
Examples of choice patterns in each condition. Individual choice pattern matrix for one participant in each condition. Numbers 1–4 in each panel indicate the choice behavior in the four training blocks. The horizontal dimension of the matrix represents participants’ sensitivity to the relative length cue, and the vertical dimension of the matrix represents participants’ sensitivity to the base rates.

The fourth prediction was that there would be more representation change in conditions where success rates are low. To quantify the change in task representation, a matrix method was used, as in the original study, to calculate the degree to which a participant has shifted in a two-dimensional representation space during the training phase of the experiment. The training problems were broken up into four blocks of 20 problems each for this analysis. The horizontal dimension of the matrix represented participants’ sensitivity to the relative length cue during each block, with the proportion of choosing the closest stick determining the position on this axis. Participants who were further from zero were more sensitive to the relative length cue. The vertical dimension of the matrix represented participants’ sensitivity to the base rates, with the proportion of choosing the successful strategy corresponding to their assigned condition. Participants who were further from zero were more sensitive to the base rate. By dividing 80 training problems into four blocks, it is possible to see how participants’ choices changed over the course of training as four points in the matrix. The rationale underlying this analysis is that choices are made based on strategies which are in turn composed from the represented task features. The change in task representation over the course of training could be characterized as the distance between each point in the two-dimensional matrix. Figure 8 shows examples for the choice patterns of four participants during the training phase.

According to the RCCL theory, the unbiased-unpredictive condition should have limited success because there were no features such as the relative length cue that were predictive of strategy success and both strategies had the same base rate of success. Therefore, there should be more representation changes in this specific condition. The results showed that the representations of the participants in the unbiased-unpredictive condition (*M* = 0.71, SD = 0.16) covered more distance than the other three conditions combined (*M* = 0.58, SD = 0.21), *F* (1, 98) = 8.09, *p* = 0.005. While the original paper only examined contrasts between the unbiased-unpredictive condition and all other conditions combined (Lovett and Schunn, 1999), we also examined the distance traveled using the biased and predictive factors (each coded as −0.5, 0.5). The interaction was not significant, and neither was the biased factor. However, a greater distance was covered when the relative length cue was not predictive than when it was predictive (*t* = −3.23, *p* = 0.002).

To verify that there was overall less success in initial strategy selection for the unbiased-unpredictive condition, whether the correct initial stick (overshoot or undershoot) was selected as the first move for the first attempt on each training problem was examined in a generalized linear mixed effect model contrasting the success rates in all other conditions to the success rate for the unbiased-unpredictive condition. The results showed that success rates in the unbiased-unpredictive condition (*M* = 0.64, SD = 0.10) were lower than the other three conditions combined (*M* = 0.69, SD = 0.10), *z* = −2.54, *p* = 0.01.

### 4.3 Individual differences results

Descriptive statistics for each of the individual difference measures are shown in Table 5. All individual differences measures were converted to z-scores prior to including them in any statistical models. To examine whether individual differences were related to initial strategy selection in BST problem solving, we first focused on the role of individual differences in initial cue use. The proportion of choosing the overshoot strategy during the pretest phase was examined using a linear mixed effects model with predictors for relative length cue and the three individual differences. The relative length cue was included to see if there was a difference in sensitivity to the relative length cue for individuals varying on any of the individual difference measures, as shown by an interaction between the relative length cue and one or more of the individual differences. No significant interactions were found, indicating no evidence for a role of these individual differences in initial cue use. However, individuals with higher inductive reasoning ability chose the overshoot strategy more often than those who were lower on the inductive reasoning measure (*b* = 0.03, *SE* = 0.01, *t* = 1.92, *p* = 0.05). There were no significant relationships between the proportion of choosing the overshoot strategy and attentional control (*b* = 0.02, *SE* = 0.01, *t* = 1.34, *p* = 0.18) or working memory capacity (*b* = 0.01, *SE* = 0.01, *t* = 1.09, *p* = 0.28). This result may indicate some differences between the overshoot and undershoot strategies where the overshoot strategy may be a bit more demanding of cognitive resources.

**Table 5:** Descriptive data and correlation matrix for the individual difference measures.

Individual differences	*M*	*SD*	Min	Max	Skew	Kurtosis	α	Correlation		
								1	2	3
1. Antisaccade score (attentional control)	27.22	6.00	9.00	36.00	−0.91	3.31	0.84	–		
2. Complex span (working memory capacity)	4.24	1.68	1.00	6.67	−0.57	2.22	0.92	0.50	–	
3. Letter series score (inductive reasoning)	0.65	0.22	0.07	1.00	−0.81	3.00	0.76	0.31	0.24	–

Antisaccade score is the number of trials for which participants gave a correct response. Letter series score is the proportion of correct trials. Descriptive statistics were calculated based on unstandardized scores. The rightmost three columns report the correlation matrix for the three measures.

Problem-solving performance during the training phase was then examined in an exploratory analysis to identify individual differences related to BST problem solving. One of the goals of this set of analyses was to identify relationships that could be explored in further hypothesis-driven research. In addition, the goal is to understand the role these individual differences may play in BST problem solving before conducting future research examining the relationship of these individual differences to strategy selection and representation change processes.

Solution time was first examined to determine if any individual differences had an influence on it. The model fit shown in Table 6 showed that inductive reasoning ability and attentional control both had a significant effect on solution time, with higher attentional control and inductive reasoning ability associated with faster solution times. In addition, being in the predictive condition also led to faster solution times.

**Table 6:** Effects of individual differences on solution time.

Predictors	*b*	SE	*t*	*p*
(Intercept)	1.67	0.02	90.80	<0.001^***^
Inductive reasoning	−0.05	0.02	−2.77	<0.01^**^
Attentional control	−0.06	0.02	−3.14	<0.01^**^
WMC	0.01	0.02	0.21	0.83
Base rate bias	−0.04	0.04	−1.21	0.23
Cue predictiveness	−0.14	0.04	−3.64	<0.001^***^
Base rate bias: cue predictiveness	0.05	0.07	0.75	0.46

All individual differences were converted to *z* scores. Solution time was log transformed. **indicates *p* < 0.01, and ***indicates *p* < 0.001.

To provide some further constraints on the mechanisms by which these individual differences influence solution time, the proportion of unique attempts and the proportion of resets that were forced resets were examined as mediators of the effect of the individual difference measures on solution time. The rationale was that measures such as the proportion of unique attempts provide indicators of how effectively participants are searching the problem space, and individual differences may be affecting this search process, which in turn impacts solution time. In this study, we defined an “attempt” as the ordered set of moves a participant used to try to solve the problem on a given trial. If the participant had not previously used that exact sequence of moves in prior attempts, then the attempt was considered “unique.” For example, consider a participant with the following set of four attempts: “A + B + A,” “C-B-A,” “B + A + A,” and “A + B + A.” There were three unique attempts and one repeated attempt. The proportion of unique attempts in this example is then 3 / 4 = 0.75. An additional example of calculating this measure with actual data is provided in the Supplementary Analysis file on OSF.

**Figure 9:**
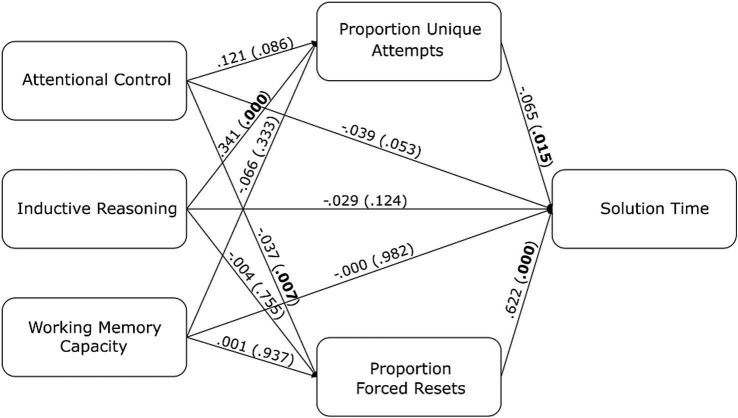
The path diagram of the dediation. Regression coefficients between all variables are labeled on each line. The *p*-values are indicated in parentheses.

The proportion of resets that were forced resets may indicate the degree to which participants are monitoring problem-solving progress on their own as compared to relying on the maximum moves built into the task interface. In addition, the condition that participants were assigned to was included in this analysis as a predictor of solution time and a predictor of the mediating variables because the problem distribution in each condition may have influenced these variables. Figure 9 displays the results of this mediation analysis excluding the condition predictors in the visualization for ease of interpretation. There was a significant path from inductive reasoning ability to the proportion of unique attempts to solution time ( b = −0.02, *z* = −2.22, *p* = 0.03), and there was a significant path from attentional control to forced resets to solution time ( b = −0.02, *z* = −2.31, *p* = 0.02).

## 5 Discussion

The goals of this experiment were to replicate a prior experiment examining how salient cues and base-rates influence strategy selection and to explore how individual differences in attentional control, inductive reasoning ability, and working memory capacity are related to BST problem-solving. The results can be summarized as follows: (1) people are sensitive to a salient feature of the task in their initial representation, (2) people are sensitive to the base rate of strategy success, (3) people tend to drop features from the task representation that are found not to be useful, (4) there are more representation changes when success rates are low, and (5) individual differences in attentional control and inductive reasoning ability are related to BST problem-solving performance, in both direct and indirect ways. The first four of these results replicate prior results (Lovett and Schunn, 1999). The prior study was one of the few to directly test the RCCL theory.

The first prediction from the RCCL theory is that salient features in a task influence the initial task representation. For BST problems, the specific prediction was that the relative length cue would influence participants’ initial strategy selection because it is the most salient cue in the task, and it will be a part of the initial problem representation. The upward linear curve in Figure 4 is an indication of individuals’ sensitivity to the relative length cue. In other words, this salient feature influences individuals’ task representations and strategy selection, which is a replication of the original finding (Lovett and Schunn, 1999). This finding is also in line with other work showing the role of salient features in strategy selection (Proctor et al., 1992).

The second prediction from the RCCL theory is that individuals will learn the success rate of each strategy. With more experience in the BST, participants will eventually learn the success rate of each strategy in the biased conditions, and they will show sensitivity to the biased base rates in their strategy selection in the posttest. Indeed, biased base rates influenced successful strategy selection, with individuals in the biased conditions selecting strategies having higher rates of success more often. This base-rate sensitivity was caused by learning in the training phase rather than any differences between condition in pretest strategy selection. One of the main reasons for developing the RCCL theory was a finding that base-rate neglect on problem-solving and decision-making tasks depends on whether the problem is presented as a text-based summary of frequencies or probabilities or whether people experience the trial-by-trial frequencies from which success rates can be learned (Bar-Hillel, 1980; Kahneman and Tversky, 1973). The current result showing sensitivity to base rates replicates the original finding (Lovett and Schunn, 1999).

The third prediction from the RCCL theory is that if current strategies have low success rates, then individuals will re-represent the task by adding or removing task features from the representation. In the BST, individuals will learn to drop the relative length cue from their task representation if it is not predictive of success. Both self-report data and behavioral data were used to examine this prediction. First, the results of the self-reports showed that individuals were less likely to use length-based cues in the unpredictive conditions relative to the predictive conditions. In addition, individuals who reported using stick length showed more sensitivity to the relative length cue than individuals who reported using the other two categories of strategies (exclusivity and other reports). These findings are consistent with the original findings (Lovett and Schunn, 1999). Choice behavior in the posttest showed that participants were less sensitive to the relative length cue in the unpredictive conditions. Sensitivity was quantified as the slope of the individual’s choice proportions against the relative length cue (e.g., the slope of the curve in Figures 4, 6), and the slopes in the unpredictive conditions became flatter from pretest to posttest. These findings indicate that individuals are reducing their use of the relative length cue when it is not useful, supporting the prediction made by RCCL that people will remove useless features from the task representation.

However, other feature-based strategy selection theories, such as the rational metareasoning (RM) framework, can also be used to interpret these results (Lieder and Griffiths, 2017). In RM’s account of strategy selection, the mapping from features to a strategy’s value is learned from experience via a set of weights between the features and the expected cost and reward of the strategy. This learning mechanism can transfer the learned weights from prior problem solving to pick effective strategies for novel problems that have similar feature values. According to RM, people can learn that a feature is not predictive of success, not necessarily because they drop the feature from the representation, but the weight between the feature and the cost and reward of the strategy could be updated to be zero. Therefore, RM can also account for these results by simply learning that the feature is not relevant to strategy selection by decreasing the weight between this feature and the reward of the strategy.

It can be difficult to ascertain whether a person’s mental representation, but the fact that the slopes in the unpredictive-biased condition were different from zero as shown in Figures 6, 7 seems more consistent with a gradual decrease in the weight of a relative length cue feature than a discrete change in representation resulting in dropping this feature from the representation as would occur in the RCCL theory. For example, if the relative length cue is removed from the task representation, then it is no longer possible to create or select strategies that rely on this feature. It will be important for future research to further test this different between the RM and RCCL theories.

The fourth prediction from the RCCL theory is that there will be more representation change in tasks when strategy success rates are low. The task representation is used to generate strategies, and the success rate of each strategy is learned by experience in the task. This learning mechanism leads to gradual changes in the estimated success rate, and these changes in turn lead to representation changes which affect the available strategies. Thus, when success rates learned for used strategies are low, people will change the task representation by adding or removing features from the task representation. In the BST, more task representation changes are expected to occur in the unbiased-unpredictive condition because neither base rates nor the relative length cue lead to high success. The results did show that there was more representation change in the unbiased-unpredictive condition than in other conditions. However, a caveat should be made concerning the conclusion that there are more representation changes in low success rates. When testing if there are more strategy changes if the success rate is low, the unbiased-unpredictive condition was compared with all other conditions as was done in the original study. But taking both the predictive and biased factors into consideration, there was no interaction between biased base rates and cue predictiveness. It may be that individuals in the unpredictive conditions have more representation changes, and there may not be more representation change in the unbiased-unpredictive condition than in the biased-unpredictive condition. A possible explanation for this result may be that the relative length cue is a salient cue in the task, and when it is not helpful, individuals tend to find other features to replace it. It may also be that the current study did not have sufficient statistical power to detect the interaction.

The method used to examine representation change as shown in Figure 8 also has its limitations. This grid method intends to examine representation change reflected in choices that are controlled by strategies. The relative length cue does seem to be a feature that could be incorporated into the task representation. However, the rate of selecting the most successful of the overshoot/undershoot solution methods is more difficult to conceptualize as a representation change. According to RCCL, success rates of generated strategies are learned via experience, but the theory does not commit to whether these are explicitly represented or learned via some implicit mechanism (e.g., reinforcement learning). Even if the base rate is explicitly represented, it would be the base rate of specific strategies and not necessarily the overall base rate of success of the initial selection of the overshoot or undershoot stick. Therefore, the base rate dimension of the grid may not reflect representation change. This analysis is also limited in that it only considers one (or two) potential task features, and if people are searching for new features that are not these features, then the analysis does not capture this variation. While this analysis does replicate the original result, there are limitations to it which make it difficult to fully evaluate this prediction of RCCL. In summary, the current study replicates the findings of the previous experiment with respect to all four predictions of the RCCL theory with some important limitations, and other theories such as RM also seem compatible with the results.

Overall, this task did introduce different strategies by manipulating features across different conditions, leading to distinct choice behaviors based on participants’ task representation, such as whether they represented the match/mismatch feature. The results supported the RCCL theory’s prediction that different strategies are generated in terms of different task representation. Moreover, initial task representations were more obvious in this task, which could help identify the representation changes. As such, the predictions made by the RCCL were tested in two relatively straightforward tasks. Despite the tasks’ simplicity, the implications of the RCCL theory are not limited to the BST. It will be important to examine the generality of this theory in tasks that go beyond the original work including tasks with a larger space of possible strategies. One challenge with examining such tasks is that it is often difficult to know exactly what strategy a participant is using when the space of strategies is larger (Moss et al., 2022).

### 5.1 Individual differences related to BST problem solving

In addition to replicating prior results, another goal of the current study was to examine relationships between individual differences in working memory capacity, attentional control, and inductive reasoning ability to BST problem-solving. In this study, we used the proportion of unique attempts, the proportion of forced resets, and solution time as measures of problem-solving performance. Problem solution time is a less granular measure of problem-solving performance that can potentially be explained by the more granular measures related to problem space exploration such as the proportion of unique attempts and forced resets in the BST task. In this task, problem solving may consist of many attempts to solve the problem, but only one solution works. Across these attempts, participants sometimes repeat an attempt from earlier. In addition, this task was programmed to force a reset on participants’ seventh move during an attempt, and the instructions for the task stated that all problems can be solved within five moves. The proportion of unique attempts and forced resets reflect how effectively people explore the problem space for each problem (Newell and Simon, 1972) and how they may be monitoring their solution attempt. Specifically, a higher proportion of unique attempts indicates that participants are more likely to maintain the traces of prior attempts and avoid repeating prior attempts. Similarly, a lower proportion of forced resets may indicate that participants are monitoring how many moves have been made to evaluate their progress toward a solution such that avoid being forced to reset.

In the regression model relating the individual differences to solution time shown in Table 6, only attentional control and inductive reasoning are significant predictors of solution time. Considering that the proportion of unique attempts and forced resets could potentially explain solution time, we also included these two measures in the mediation model as mediators. Individual differences in attentional control and inductive reasoning ability influenced solution time but were mediated by the proportion of forced resets and the proportion of unique attempts, respectively. Higher inductive reasoning ability was associated with a higher proportion of unique attempts, leading to faster solution time. One interpretation is that participants are reasoning about the failure of past attempts to plan the next attempt, which helps to constrain search in a manner that reduces the chance of repeating an earlier attempt.

Participants with higher attentional control had fewer forced resets, leading to faster solution time. This mediation can be explained by the fact that attentional control resources are being used to monitor the number of moves made so that reaching a forced reset is less likely. Attentional control has been discussed as the ability to control the information that gets maintained in working memory in the face of distraction (Unsworth, 2016; Unsworth et al., 2021). In the antisaccade task, this control allows the goal of attending to the opposite side of the screen to be maintained and influence action selection in the face of the prepotent response to move one’s eyes to the blinking distractor. In the BST, this process may allow for a move counter to be maintained while also executing planned moved and planning future moves.

Finally, there was no effect of working memory capacity on problem-solving measures. This result is not aligned with other work, which found that individual differences in WMC are related to the ability to retrieve previous task experience which then contributes to task performance (Mattarella-Micke and Beilock, 2010; Wiley and Jarosz, 2012). One possibility for the nonsignificant result is that working memory capacity is correlated with attentional control, and the latter accounts for the effects on problem-solving performance (Ash and Wiley, 2006; Engle and Kane, 2004; Kane et al., 2001). As such, it is reasonable to suggest that solution time is not influenced by the unique variance of working memory capacity that is not shared with attentional control and inductive reasoning. As expected, all the task measures were somewhat correlated with the antisaccade and complex span measures sharing 25% of their variance. As shown in a supplemental material, when working memory capacity alone is used to predict solution time, then there is a significant relationship, but if either of the other two individual differences are included, then this relationship is not significant. Therefore, the lack of a relationship with working memory capacity in the current study could be attributed to variance better accounted for by the inductive reasoning and attentional control measures.

The interpretation of the individual differences is also limited by the use of a single task to measure these three related latent constructs. There is always the concern that the correlations we observed could be due to task variance unassociated with the latent construct. In addition, the status of attentional control as a singular construct is very much an open question. We selected the antisaccade task because of its reliability and that it generally loads highly on an attentional control factor in other research (Draheim et al., 2021). Traditional tasks that have been used to measure attentional control suffer from low reliability and do not correlate with each other (Hedge et al., 2018; Rouder and Haaf, 2019). There are different proposals on how best to measure attentional control given these concerns including relying on accuracy-based measures such as the antisaccade rather than on differences in response times such as in a Stroop task (Burgoyne et al., 2023; Draheim et al., 2021). Researchers examining the related construct of cognitive control have also noted that there are multiple mechanisms or dimensions by which individuals could adjust to task demands (Frömer and Shenhav, 2022; Ritz et al., 2022), but it remains to be seen whether it is possible to measure reliable individual differences along these dimensions.

Although the present results are consistent with the original findings, they also raise some new questions. For example, we found that participants showed sensitivity to the relative length cue in the pretest, but there was also a relationship between inductive reasoning ability and use of the overshoot strategy. It is not clear why this relationship exists. In the BST, we can consider the overshoot strategy as subtraction and the undershoot strategy as addition. The preference for undershoot could be induced by the preference for addition rather than subtraction (Adams et al., 2021). Inductive reasoning, a core component of fluid intelligence, is also closely tied to math performance (Kinshuk et al., 2006). And using a subtraction procedure has been found to be more difficult than addition (Barrouillet et al., 2008; Campbell and Xue, 2001; Kamii et al., 2001). People with higher inductive reasoning ability might be better at using subtraction or more willing to engage in the reasoning required to plan a subtraction solution, which can be the reason why they choose overshoot strategy more.

It is noted that the individual difference analyses in the current paper were exploratory analyses, and it was not feasible to examine the relationships between individual differences and base-rate learning in only a subset of the conditions, such as in the biased-unpredictive condition. Examination of individual differences in the rate at which people shift toward more useful task representations and strategies with that kind of comparison was not possible in the current study given that each cell of the design had only 25 participants. Importantly, exploring the relationships between individual differences and problem-solving performance should provide a basis for examining how these individual differences play a role in strategy development and selection in future research using BST problems to explore strategy selection. Verifying that the results of prior research could be replicated was a prerequisite to conducting a higher-powered study examining the role of individual differences. This future work would also be better able to address the question of whether strategy success estimates are maintained and updated explicitly or implicitly by examining individual differences related to adapting to base rates.

To conclude, in spite of some limitations, we have tested the predictions made by the RCCL theory and replicated the original findings from Lovett and Schunn (1999). However, the RCCL theory needs further development to test more specific predictions, and some of the results may be better explained by other strategy selection theories such as RM. Individual differences were also found to be related to BST strategy use and problem-solving performance. Based on the present findings, future research can focus on exploring strategy preferences and establishing a more thorough understanding of how individual differences are related to strategy development and selection.

## Data Availability

The datasets presented in this study can be found in online repositories. The names of the repository/repositories and accession number(s) can be found below: https://osf.io/8w4kd.
